# An Ill-Fated Hospital

**Published:** 1901-03-30

**Authors:** 


### An Ill-Fated Hospital.

Fate is not kind to the National Hospital for the Paralysed. At the meeting on Saturday the Governors instituted an enquiry into all matters in dispute between the medical staff and the Board. It was remarkable after more than a year's contest that the Board had no speaker to put forth their case. Nothing but ancient history was forthcoming from Mr. Russell, and when the solicitor started by saying he would justify the Board, he not only failed to do so but threw up the sponge. In such circumstances the Governors promptly realised that the Board had never actively interested themselves in the matter at all. They were convinced that it had been throughout a battle between the solicitor, Mr. Bower, acting through Mr. Rawlings the secretary-director, and the medical staff backed by the medical profession. How could the Board have been so weak as to thus abrogate their functions and sacrifice their authority? But worse even remained! Gentlemen of position will hesitate and even refuse to act upon the committee when they learn that the evidence which may be given before them is not to be published. The enquiry, if great care is not exercised at the start, may therefore be unreal and ineffective! What man of reputation and standing will consent to sit upon a Committee, the report and findings of which will not be supported by evidence? Mr. Bower declared at Saturday's meeting that the evidence should not be published, and it was so decided. Is the enquiry, then, to be private, and are the press to be excluded from it? If so, what good can be hoped for from such an enquiry so conducted, where material facts may be withheld from the Governors, the profession, and the public? What authority will attach to a report and findings which are unbacked by evidence? What eminent lawyer, experienced physician or surgeon, financial expert, experienced hospital administrator, or other person of character, is likely to risk his reputation by allowing himself to be made the shuttlecock of rivals engaged in bitter personal conflict, as he will do if he serves on this Committee, his decisions being divorced from the evidence upon which they are based. Mr. Melville Green's attitude on this point is to us unaccountable. We can only suppose that his apparent support of Mr. Bowers' policy of non-publication of the material points of the evidence (they jointly voted down the publication of the evidence before the enquiry at Saturday's meeting) must be based upon a serious misapprehension. As matters stand at present, the committee of enquiry, handicapped as it will be by the secrecy of its proceedings and the suppression of the evidence given before it, is likely to fail, and deserves to fail in securing peace and prosperity for this valuable but ill-fated hospital. We would urge everybody invited to serve on the committee to insist upon a public enquiry or to refuse to take part in it. Any other course is calculated to destroy and not to rehabilitate the credit of the National Hospital for the Paralysed and Epileptic. To suppress the evidence will probably destroy the hospital. Those whom the gods wish to destroy, they first make mad! Is ineptitude of procedure by the Board to be followed by the mad policy of suppression of the evidence given? We sincerely hope not!

### The Friendly Societies and the Wage Limit.

It cannot be said that the medical profession has figured very well in the discussion on the wage limit at the Conference of Friendly Societies recently held at Birmingham. From the beginning of the negotiations as to the establishment of a conciliation board, it had been state clearly enough by the representatives of the Friendly Societies that they would have nothing to do with any wage limit, yet at the meeting which took place in February of this year between a Committee of the British Medical Association and representatives of the Societies this very subject was brought forward as the all-important question by the President of the Association. In the end a long and perhaps not altogether wise statement was drawn up and signed by Dr. Elliston, and submitted to the Conference in Birmingham. But it was not to be expected that a mere statement of the views generally held of by medical men on the subject, made by a body which confessedly had neither the right to speak for the medical profession, nor the power to ensure the performance of any promise it might make on their behalf, would have any weight with bodies which had already stated that they had made up their minds. No one need then feel surprised that the Conference unanimously resolved that "with regard to the question of wage limit the Committee of the British Medical Association be informed that this National Conference of Friendly Societies cannot agree under any circumstances to the adoption of such limit."

The fact has to be recognised that the Friendly Societies have a representative organisation, and that the medical profession has nothing of the sort. The General Medical Council has given its blessing to the suggested Conciliation Board, but it is limited by its statutory powers, and can take no action in the matter; while the British Medical Association at the outside only represents its own members, and even for them can offer no promises and can make no threats. The key to the unsatisfactory position of the club doctors is probably to be found in the statement made by one speaker at the Conference to the effect that "whenever there was a vacancy for a doctor in his part of the country there were about ten applicants," and that "so long as that state of things existed he did not think they need be afraid of the medical profession." That is the exact truth. If we had been able, as a united profession, to send authorised representatives who could have said to the Conference, "If you will not give way on this matter we will withdraw your medical officers," and if our organisation had been such that we could have given effect to the threat, the Friendly Societies would have been complaisant enough. But such was not the case.

We do not say that any such trades-union arrangement appeals to our professional sympathies—far from it. But we do say that if it becomes a matter of conferences and boards of conciliation the medical profession will meet with nothing but humiliation until it has some such power; and that until then it had far better eat its humble pie in private than before so many witnesses. All such boards and conferences will be harmony itself as long as the medical profession yields each point; but directly it makes a stand it must be beaten so long as we are unable to act together and enforce our decisions. Argument is useless; and as things are at present there is this great evil in boards of conciliation and all similar bodies, that their decisions are sure to be taken as bearing an official stamp, and as having an authority which they by no means possess. It is perhaps a small matter that a doctor here and there should kow-tow to the officials of his little club. It is bad enough, but after all he is an insignificant person, and nothing that he does can be taken as a precedent. It is quite otherwise, however, when resolutions derogatory to the profession are passed, as they surely will be passed if conciliation boards come into vogue, by bodies containing medical men of well-established position. Such resolutions, however little binding they may be, will be taken by the public as authoritative, and great harm must result. The profession must make up its mind either to go on in the way of its fathers—to stand on its dignity and do nothing—or to organise and do something. One or the other. Above all things it must not palaver unless it knows that it can act.

### Drink and the Barmaid.

In our efforts to control intemperance we may either attack "the drink" itself, a crusade noble, possibly, but quixotic, or, with the Bishop of Winchester, we may adopt the humbler but more practical method of strengthening the powers of the law for the suppression of certain generally recognised evils connected with the trade in alcohol. Among those evils may we not place the barmaid? That she is a "charming creature" we are, of course, ready to admit, and that she is often an honest hard-working and much over-worked woman we not only admit but know. But all the same she is responsible for much evil. No one who is compelled by unkind fate to live much away from home can help observing to how large an extent the barmaid is responsible for the initiation of drinking habits among young men, and when one considers how many young fellows there are in all commercial towns who live away from home and hardly speak to any women except waitresses and barmaids, we cannot wonder at it. Barmaids are the syrens who lead the young to drink. Of that there can be no doubt, and the question is whether the purveyors of alcohol should be allowed to use up such a mass of maidenhood as is annually sacrificed to the trade, merely for the sake of giving additional attractiveness to the drink they sell. Barmaids as a class are much to be pitied. They have to work hard, they have to stand long hours, and to suffer all the evils which long standing causes in women, while constantly before them are the bottles and the taps from which temporary comfort from these miseries can be obtained. Can we wonder that some give way to the temptation, and that the occupation of barmaid is an unhealthy trade. As to the morality of the work we will not say much. That many barmaids are as good as most people goes without saying, as it also does that many, and especially the younger of them, are exposed from week-end to week-end to temptations to which no woman —or girl as she often is—ought to be exposed as part of her business-life. No woman ought to be "put in the shop window," so to say, as part of the attraction of the shop, as is practically done with barmaids. That the work of barmaids is far more full of risks, and demands inspection far more than many an industry which is ticketed as "dangerous," we are quite convinced; and when to that we add the danger to the public which accrues from the employment of, often, physically-attractive women as lures to drinking, and as a means of encouraging drinking habits, we think we are on the right side in saying that if barmaids cannot be at once abolished, a very heavy tax on their employment and a very careful inspection of the conditions under which they are employed would, to say the least of it, make for temperance.

### A Spending Department.

The Metropolitan Asylums Board appears to be the very type of a spending department, for it is under but little direct control by any of the various bodies by which its members are appointed, while the interests of these bodies are so varied as to make it very improbable that any consistent effort at economy would obtain support. There are a few members on the Board who undoubtedly stand for economy, but it is to be doubted whether either they or their measures are very popular, while on the other hand it is never very difficult for the open-handed ones to draw to themselves a wide support when they can point, as indeed they often can, to good work well done as the result of the expenditure which they have advocated. The constantly increasing expensiveness of all that is undertaken by the Board is however a matter which must give rise to anxiety, and all the more so when we bear in mind how difficult it is to control. Speaking at a recent meeting of the Board the chairman of the financial committee showed that, except in regard to the training ship, every class of institution under their management and every group of expenditure except maintenance charges showed an increase, notwithstanding that the total number of inmates was less than before. No one can deny, nor indeed should we think that anyone would care to deny, that some of the work done by the Board is performed in an admirable manner. But whether all the work is done in an equally admirable manner, and whether the desire to economise manifests itself in equal degree in the various departments under the control of the Board, is another question.

